# Macroscale optimal size of ICM vesicles regulated by quantum design principle in LH2 structure

**DOI:** 10.1016/j.bpj.2025.06.004

**Published:** 2025-06-07

**Authors:** Ying Zhang, Qianjin Chu, Luchao Du, Yugui Yao, Hailong Chen, Peng Wang, Jianping Zhang, Mingqing Chen, Lingfeng Peng, Yuxiang Weng

**Affiliations:** 1 Laboratory of Soft Matter Physics, Institute of Physics, Chinese Academy of Sciences, Beijing, China; 2 University of Chinese Academy of Sciences, Beijing, China; 3 Institute of Physics, Chinese Academy of Sciences, Beijing, China; 4 Beijing Institute of Technology, Beijing, China; 5 Songshan Lake Materials Laboratory, Dongguan, China; 6 Renmin University of China, Beijing, China

## Abstract

The photosynthetic bacterial light-harvesting antenna complex 2 (LH2), consisting of ring-like bacteriochlorophylls aggregates, constitutes an optimal excitonic structure for efficient energy transfer. Any distortion from this structure would cause efficiency losses. When adapted to low-light growing conditions, LH2-embedded membranes form vesicles to enhance light capture, albeit at the expense of curvature-induced LH2 deformation. Therefore, evolution should optimize vesicle sizes for overall light utilization efficiency. To unveil this optimization strategy, LH2 was assembled onto silica nanoparticles of a wide size region to simulate LH2 deformation, which was characterized by the B850 lifetime both theoretically and experimentally. We found that LH2 was undeformed only within the size range of 50–80 nm, akin to vesicle sizes observed in bacteria, suggesting that vesicle size optimization follows the LH2 structural design principle.

## Significance

Our findings reveal the stiffness and robustness of the quantum principle-designed structure of LH2, ensuring efficient energy transfer even in the presence of environmental variations, including curved membranes. The maintenance of this exceptional structural design necessitates the optimization of vesicle sizes. This study exemplifies quantum effects in biology at various scales, spanning from the sub-10 nm LH2 protein complex to 50-nm membrane vesicles and extending to the overall intracytoplasmic membrane morphology within bacterial cells.

## Introduction

The efficiency of photosynthesis relies on ultrafast energy transfer from the photosynthetic antenna to the reaction center (RC) for charge separation within a picosecond timescale (1). These processes are vital for the organism’s viability and are regulated and optimized to harvest solar energy and facilitate subsequent energy transfer. It is expected that the macroscale architectures of certain organelles in photosynthetic organisms are influenced by the microscale structures of the light-harvesting antenna complexes (LHs) through a process of stressed coevolution. Notably, some LH structures adhere to the quantum design principles. For instance, in higher plants, recent cryoelectron microscopy (cryo-EM) structures of quenched and unquenched major light-harvesting complexes of photosystem II (LHCIIs) show that LHCII functions as a protein-activated quantum switch by tuning the distance between a main quenching pigment pair of Lut1-Chl612 around a critical separation of 5.6 Å, leading to a transition between an efficient light collection state via Förster energy transfer mechanism (Lut1(S_2_ state) → Chla612(Q_y_ state)) under low light intensity (>5.6 Å) and a photo-protection state via the Dexter energy transfer mechanism (Chla612(Q_y_ state)→Lut1(S_1_ state)) for ultrafast energy dissipation known as nonphotochemical quenching (NPQ) under high light intensity (<5.6 Å) (2). In algae, the core antenna allophycocyanin (APC) represents a simple excitonic dimeric system of exciton-vibration coupling. This system achieves quantum phase synchronization of the resonant vibrational collective modes, acting as a quantum version of a classical Huygens pendulum pair, thereby safeguarding long-lasting coherences against environmental noise for efficient energy transfer (3,4). In photosynthetic bacteria, the special and tight packing of bacteriochlorophyll molecules into two pigment rings in the peripheral light-harvesting complexes (LH2s) is based on the rational exciton-vibration coupling that utilizes the spontaneous fluctuations associated with the quantum motion of the nuclei, resulting in thermodynamic stabilization, directional energy flow, and high transfer efficiency (5). Moreover, the nanoscale design principle governing LH2 optimal natural sizes (8- to 10-fold symmetries) mitigates disorder through the cooperative action of hydrogen bonding and quantum delocalization (6).

To survive under changing light conditions, photosynthetic organisms employ adaptive strategies such as adjusting the ratio between LHs and the RCs, modifying their spatial arrangement, and adapting the morphology of the photosynthetic membrane (7,8,9,10). For example, photosynthetic bacteria synthesize intracytoplasmic membranes (ICMs) to increase their intracellular membrane area available for light absorption and utilization, particularly under conditions of low light intensity (7,9,11,12). The ICM consists of photosynthetic units composed of RCs encircled by LH1s and loosely associated with LH2s (13). These specialized membrane proteins accommodate pigments with finely tuned binding site and spatial orientation to ensure efficient and directional energy and electron flow (14). The morphologies of ICMs, including vesicles, tubules, and stacked lamellar membranes, vary depending on the bacterial species and growth conditions (10,15).

**Figure 1:**
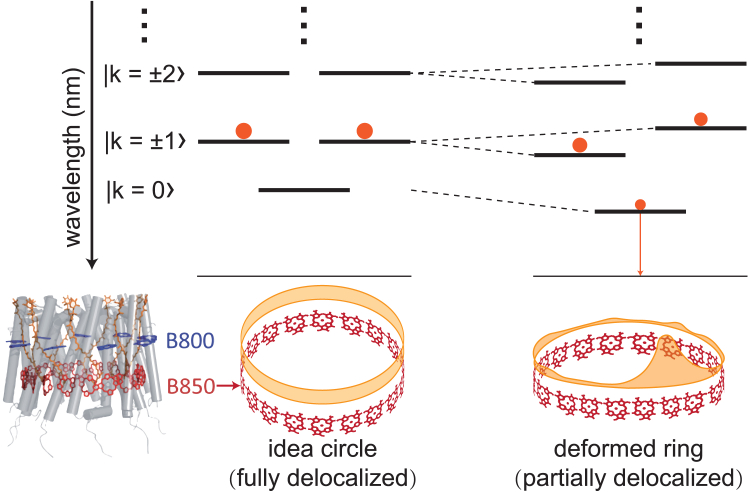
Schematic diagrams illustrating LH2 crystal structure and excitonic energy levels denoted by k and exciton delocalization of the B850 pigment ring. The filled orange circles represent the population distribution of a given excited state. The red BChl *a* ring symbolizes the B850 ring, while the yellow band signifies the extent of exciton delocalization across the B850 ring (23,24). The inserted schematic diagram of LH2 structure is reproduced from (34) with permission from the Royal Society of Chemistry. In the case of a perfectly symmetric B850 ring, the excitation energy is initially delocalized across all 18 BChl *a* molecules. Any departure from this situation results in energy level splitting of the degenerated states and the localization of excitation energy on a few BChl *a* molecules. The lowest excitonic state becomes partially optical-transition allowed.

In particular, vesicular structures found in *Rhodobacter* (*Rba.*) *sphaeroides*, which are among the smallest organelles for photosynthesis, with an abundantly observed size distribution of 50–80 nm, have been of great research interest (7,9,16,17). Under low light intensity, specialized paracrystalline LH2 domains (10) and ICM vesicles (7,9) grow to enhance photon capture. Conversely, under high light conditions, fewer ICM vesicles are observed, accompanied by a decreased ratio between LH2 and RC (7). These vesicles serve as excellent model systems for studying light-harvesting and energy transfer mechanisms due to their increased surface area per volume (15) and optimized bio-energetic properties of the involved protein complexes (18). As stated above, LH2, the major component in ICM vesicles, represents a highly optimized electronic structure of pigment aggregates for energy storage and transfer following the quantum principles (14). The structures of LH2 have been resolved at atomic resolution using X-ray crystallography (19) and cryo-EM (20,21). LH2 is composed of 7–10 heterodimers, with each heterodimer consisting of an α and a β transmembrane polypeptide. The assembly of heterodimers produces a symmetrical, hollow, cylindrical structure where each heterodimer hosts three noncovalently bound bacteriochlorophyll *a* (BChl *a*) and one carotenoid molecule. Within this structure, two BChl *a* are held parallel to the membrane normal, forming a pigment ring that absorbs light at 850 nm (B850 ring), while the third BChl *a* is oriented nearly perpendicular to the membrane normal, creating a pigment ring that absorbs light at 800 nm (B800 ring) (19). Energy transfer occurs within and between the two rings, facilitated by the alignment of BChl *a* molecules at specific distances and orientations, providing an optimal geometry for energy transfer and enabling efficient energy transfer through excitonic delocalization (22), as depicted by Fig. 1. An idealized B850 ring in the absence of site energy disorder and structure deformation, based on the excitonic theory, features a lowest excited state (k=0 state in Fig. 1) that is optically forbidden, allowing excitation to be preserved, serving as an energy storage ring. Moreover, the B850 ring is an efficient energy donor with near-perfect quantum efficiency for LH2-LH2 and LH2-LH1 inter-complex energy transfer (22,23). When considering the flexibility of the protein and the site energy disorder, the excitonic state may not extend over the entire LH2 ring, leading to partial optical-transition-allowed states, as shown in Fig. 1 (23,24). Even for a ring-symmetry LH2, van Grondelle et al. show that disorder can break down the exciton delocalization over the ring and cause the lowest state to become dipole-transition allowed, leading to the superradiance in LH2 (25). Moreover, the ideal structure of the LH2 ring can undergo further elliptical or tilted circular distortions due to various environmental factors, promoting the decay of excitation energy through various processes (26,27). The quantum efficiency (η) of excitation energy transfer (EET) from LH2 to RC depends on the decay rate of B850 in LH2 ($k_{decay}=1/\tau$) and the trapping rate of RC ($k_{trap}=1/\tau_{trap}$), given by $\eta =k_{trap}/\left(k_{trap}+k_{decay}\right)$ (28). Obviously, the lifetime of LH2 (τ) has a significant effect on η. For instance, when the lifetime of B850 at room temperature is about 1.3 ns and the trapping time is about 100 ps (1), the EET efficiency is around 93%. If the lifetime of LH2 is reduced to 0.7 ns, then η would drop to 87%. Studies on the deformation of LH2 indicate that the presence of a curved membrane and the surrounding electrostatic environment of vesicles may cause LH2 to deviate from its optimal structure and promote the decay rates of B850, causing energy loss and the decrease of η (26,27,29). Notably, although increasing the whole antenna size enhances light absorption, it may also prolong trapping times due to increased exciton migration pathways (30), potentially reducing η. Some studies suggest that decreasing the antenna size, as a direction for optimization, can enhance photosynthetic rates (31,32). Recent work also highlights that interpigment charge-transfer state couplings contribute to excitonic-site energy shifts and disorder of the B850 ring in LH2 (33), particularly that the k=0 excitonic state is more red shifted and more emissive. All these facts suggest that the vesicles formed in low light intensity can enhance the light-harvesting ability but at the cost of a reduction in energy transfer efficiency.

This raises a fundamental question regarding the evolutionary process, i.e., to ensure the total light conversion efficiency of a photosynthetic cell, especially under low-light-intensity stress, how do photosynthetic bacteria strike a balance between having a larger photosynthetic membrane area (achieved through smaller vesicle sizes) and maintaining a higher efficiency of energy transfer (minimizing LH2 protein deformation at larger vesicle sizes) to enhance the total light conversion efficiency of a photosynthetic cell?

Earlier efforts have been devoted to elucidating the size of the self-assembled ICM vesicles, as well as the efficient energy transfer mechanisms of LH2 and LH1-RC (9,35,36). A kinetic model has suggested that the formation of ICM vesicles in response to light adaptation of bacteria is a spontaneous optimization process balancing structural integrity and robust energy conversion (36). However, the impact of LH2 deformation inflicted by curved membranes and electrostatic interactions on energy transfer efficiency remains elusive. Although the optimal design of nanoscale LH2 structures has been explored in relation to quantum effects (6), we wonder whether the organelle-scale ICM vesicle size is still optimized and regulated by quantum principles, i.e., how quantum-dynamical phenomena at the nanoscale can provide a selective advantage to an overall organism (37). To address this question, we explored the excitonic-state dynamics of LH2 assembled onto charged silica nanoparticles of varying sizes (LH2@SiO_2_). We examined the lifetime of LH2 on the curved, negatively charged surface of silica nanoparticles ranging from several nanometers to 550 nm. The research was structured as follows: 1) the vesicle was analogously considered as an incompressible liquid sphere, with its mechanical properties simulated by a silica sphere. This assumption was supported by our observation that the changing trend of curvature-modulated deformation of LH2 was comparable between LH2 embedded in liposomes and LH2 adsorbed on silica surfaces of similar sizes achievable for the liposome. 2) In-plane and out-of-plane deformation of the LH2 ring was quantitatively derived using classical mechanics. 3) The radiative decay rate, expressed in terms of oscillator strength, of the deformed LH2 ring was calculated quantitatively through excitonic theory. This step aimed to establish a relationship between LH2 ring deformation and the corresponding excited-state lifetime. 4) The experimentally measured LH2 lifetime on nanoparticles against the size of silica nanoparticles was compared to the theoretical relations, followed by a discussion and summary of the findings.

## Materials and methods

Nanoparticles of specific sizes were refined from the commercial colloidal solutions by ultracentrifugation (38). The particle sizes were analyzed using a JEM 2010 transmission electron microscope (JEOL, Peabody, Massachusetts). The transmission electron microscope images were used to statistically evaluate the average sizes and corresponding size distributions of the nanoparticles (38). The LH2 samples used in the study were purified from *Rba. sphaeroides* 2. 4. 1. The procedures for LH2 preparation, purification, and assembly onto nanoparticles have been described previously (26,29,34). The concentration of LH2 was adjusted to OD_B850_ = 1.0 cm^−1^ in 20 mM Tris-HCl buffer (pH = 8) containing 0.1% LDAO. The preparation of LH2 in liposomes, named LH2-Ls, was the same as reported previously (39). Briefly, 3 mg L-α-phosphatidylcholine (Sigma-Aldrich, St. Louis, Missouri) was mixed with 6 mL 20 mM Tris-HCl buffer solution (pH 8.0) and 100 μL of 30% LDAO to form a homogeneous solution. A 100 μL LH2 stock solution (OD_850_ = 34.98 cm^−1^) and 450 mg Bio-Beads SM-2 (Bio-Rad, Hercules, California) were successively added to the above mixture. The resulting mixture was shaken at different temperatures (temperature-controlled shaker) to control the rate of adsorption of detergent by bio-beads and hence the size of self-assembled vesicles: small size (60 nm) taken 45 min at 25°C, middle size (70 nm) 120 min at 15°C, and large size (100 nm) 180 min at 5°C. After removing the bio-beads by filtration, vesicles were prepared from the resuspended lipid films by sequential extrusion through polyether sulfone filter membranes with a pore diameter of 220 nm 10 times. The protein/lipid molar ratio was approximately 1:4000. The morphology of liposome samples was characterized by cryo-EM (200 kV, Thermo Scientific Glacios 2). To obtain the LH2s within the photosynthetic membrane, the photosynthetic bacteria cells were cultured under low-light-intensity conditions of 10 W/m^2^ to ensure a high content of LH2. The resulting membrane primarily consisted of LH2, as confirmed by its UV-visible absorption spectrum (Fig. S1 *D*). The fluorescence decay kinetics in Fig. S1 *C* were measured by the time-correlated single-photon counting system (Edinburgh Instruments, Livingston, United Kingdom) obtained for the LH2 excited state in photosynthetic vesicles and in membrane fragments. The vesicles were prepared by disruption cells with a French press, and the membrane fragments were prepared by an ultrasonic crusher. Micrographs of the LH2-embedded membrane were taken with a JEM100CXII electron microscope (JEOL).

The excited-state lifetimes of B850 for LH2 on silica nanoparticles (LH2@SiO_2_) were measured by home-built femtosecond time-resolved transient absorption (TA) measurement. The detailed information has been reported in a previous work (29). TA measurement is insensitive to the concentration of silica (Fig. S2), and the silica colloidal concentrations were kept at 2.0 g/L. For femtosecond TA measurements of LH2-Ls, the HARPIA-TA spectroscopy system (Light Conversion, Vilnius, Lithuania) was employed. The femtosecond laser (PHAROS, Light Conversion) centered at 1030 nm with a pulse repetition rate of 100 kHz and a pulse width of 190 fs was divided into two beams. One was sent to ORPHEUS-HP (Light Conversion) to produce tunable excitation pulses around 800 nm and the excitation energy before the sample was attenuated to 0.4 nJ. Another beam was focused into a 1-mm-thick sapphire crystal, generating supercontinuum white light spanning from 500 to 1000 nm as the probe pulsed. The relative time delays between the pump and probe pulses were modulated by a mechanical delay stage. The fluorescence quantum yield was measured with the absolute photoluminescence quantum spectrometer (C11347, Hamamatsu Photonics, Hamamatsu City, Japan).

## Results

### Justification of using silica nanosphere as a substitute for vesicle

Currently, it is challenging to systematically investigate the deformation of LH2 on vesicular ICM due to the difficulties in ensuring uniform and controllable sizes of artificial vesicles (liposomes) within the desired range, typically ranging from tens of nanometers to submicrometers. As an alternative approach, silica nanoparticles are employed as substitutes for vesicles to simulate the behavior of LH2s on vesicular ICM in a controlled and manageable manner. These nanoparticles can be prepared to have a rather uniform size and a spherical shape (Fig. S3) with known surface charge density (38).

**Figure 2:**
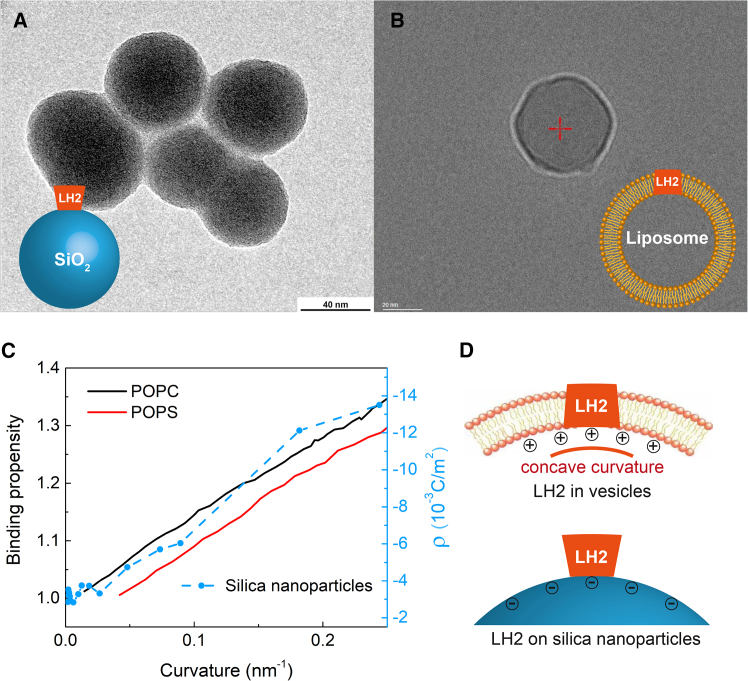
Images for silica nanoparticles and phospholipid bilayer vesicles (containing LH2) and diagrams showing the resemblance of size-dependent surface charge densities between the vesicles and silica nanoparticles. (*A*) Transmission electron microscopy (TEM) image for silica nanoparticles of typical size with a diameter of 60 nm, and the inset graph is a diagram of LH2 attached on charged silica spheres. (*B*) Cryo-EM image of LH2 reconstituted into lipid bilayer vesicles (LH2-Ls) with a lipid/protein molar ratio of 4000:1, and the inset is the schematic representation of LH2-L. (*C*) Comparison of the surface charge density on the curved phospholipid bilayer membranes reflected by the binding propensity of Ca^2+^ with the surface charge density of silica nanoparticles of varying curvature. The black line is the binding propensity of Ca^2+^ to phosphatidylcholine (POPC) lipid membrane. The red line is the binding propensity of Ca^2+^ to phosphatidylserine (POPS) lipid membrane. The binding propensity was calculated based on molecular dynamics simulation by Yesylevskyy et al. (40) The blue dotted/dashed line is the surface charge density (ρ) of silica nanoparticles against varying curvature (38). (*D*) Schematic representation of the charge distribution on the inside of the phospholipid vesicles and the surface of the silica spheres and the orientation of LH2.

To validate the nanoparticle-vesicle substitution approach, we systematically compare the force profiles acting on LH2 in both systems (vesicle-embedded versus nanoparticle-adsorbed configurations). First, when the size of vesicles or silica nanoparticles is significantly larger than that of LH2, it can be shown that the shear forces on LH2 in both systems are formally proportional to the surface curvature (1/R). The fundamental origins differ: in vesicles, the dominant shear component stems from the Young-Laplace pressure difference (ΔP=2γ/R) between the membrane and the cytoplasm interfaces induced by the surface tension (γ) (see supporting text, for complete derivations); on nanoparticles, the shear force is derived from curvature-dependent Coulombic interactions. Then, we also compare the relationships between surface charge density and curvature in the two systems. The surface charge of silica particles is determined by the protonation or deprotonation of silanol functional groups, which depends on the local solution pH, ionic strength, and particle size (38). Larger silica particles with smaller curvatures exhibit a higher propensity for proton binding, resulting in a reduction in negative charge density on the particle surface. For the intracellular membrane, Yesylevskyy et al. show that Ca^2+^ ion exhibits a preference for binding to the concave membrane monolayers with higher curvature, according to the calculated binding propensity of Ca^2+^ ions to lipid bilayers as a function of curvature by the molecular dynamical simulation (40). The enrichment of cations at the concave membrane with larger curvature results in a higher positive surface charge density. Other works also supported the observation of a significant increase in membrane surface positive charge density with large concave curvatures (18,41). Overall, the relationship between the surface charge density of the concave membrane side and the vesicle size follows a similar trend to the size-dependent surface charge density of silica nanoparticles, justifying the use of charged silica nanoparticles as a suitable model for investigating the curvature-induced LH2 deformation in ICM vesicles, as illustrated by Fig. 2.

Additionally, from a conceptual perspective, the cytoplasm within the vesicle is typically modeled as an incompressible fluid (42,43), suggesting that the ICM vesicles can be regarded as hard spheres similar to silica nanoparticles. As a result, the force profiles acting on LH2 attached to charged silica nanoparticles closely resemble those in ICM vesicles. We therefore conclude that nanoparticles serve as a valid substitute for vesicles in inducing LH2 deformation.

Further, we investigated the curvature-induced lifetime changes of LH2 embedded in liposomes (LH2-Ls) of several sizes. Due to challenges in preparing uniformly sized liposomes in the broader size range, the LH2-L samples were limited to a specific size range. The lifetimes of LH2-Ls sized at 60 ± 40, 70 ± 45, and 100 ± 60 nm were measured, yielding respective lifetimes of 0.77, 0.93, and 0.50 ns. The sizes of the LH2-L samples were characterized using cryo-EM imaging techniques (Fig. 2 *B*) and dynamic light scattering (Fig. S4 *A*). The bleaching kinetics at ∼860 nm and the fluorescence and absorption spectra of LH2-Ls are also given in Fig. S4. It is shown that the fluorescence peak for LH2-Ls is red shifted and broadened compared to free LH2, indicating the deformation of LH2. This observation is consistent with a previous spectroscopy study comparing free LH2 with LH2 adsorbed on SiO_2_ nanoparticles (29) and those embedded in the vesicles with an averaged diameter of 39.4 nm (44). To further support the curvature-induced LH2 deformation by a membrane, we measured the fluorescence decay kinetics of LH2 in photosynthetic vesicles and flat membrane fragments from *Rba. sphaeriodes*, as shown in Fig. S1. The results clearly demonstrate that the lifetime of LH2 in curved membranes (0.31 ns) was shorter compared to that in the flat membrane fragments (0.41 ns). This provides additional evidence for the influence of membrane curvature on LH2 deformation.

### Derivation of particle-size-dependent LH2 protein deformation

**Figure 3:**
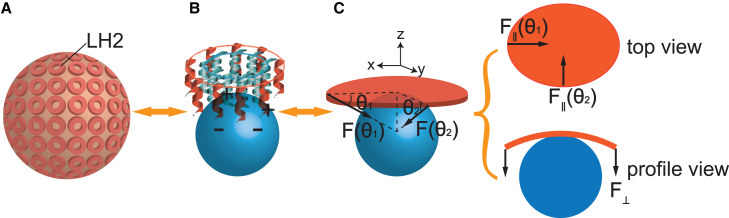
Schematic diagrams depicting LH2 deformation on charged silica nanoparticle surface mimicking the ICM vesicles surface. (*A*) Model of LH2 embedded in the membrane of a vesicle. (*B*) Model of LH2 adsorbed on a negatively charged silica nanoparticle via Coulombic interactions. (*C*) Schematic representation illustrating the induced deformation of LH2 as an elastic plate by nanoparticles, including in-plane elliptical deformation (*top view*) and out-of-plane bending deflection (*profile view*). $F_{∥}$ and $F_{⊥}$ denote the in-plane and out-of-plane components of the Coulombic forces, respectively. $\theta_{1}$ and $\theta_{2}$ are defined as the angles between the *xy* plane and the line connecting the center of the sphere and the endpoint of the long axis or short axis of the LH2 ellipse, respectively.

The cryo-EM structure of LH2 from *Rba. sphaeroides* indicates that its dimension can be approximated as a cylinder with a diameter of 76 Å and a height of 66 Å (21). Neutron diffraction studies have revealed that the cavity of the cylinder is filled by detergent or a lipid core (45), leading to the visualization of the topological structure of LH2 as an elastic plate. In light of these structural insights, we have employed plasmonic surface-enhanced Raman spectra to investigate LH2 adsorbed on Au core silica shell (Au@SiO_2_) nanoparticles, which confirmed that LH2 adheres to the nanoparticle surface, with the positively charged cytoplasmic side (N-terminal) of LH2 oriented toward the negatively charged nanoparticle surface (46). Besides, we have determined the surface charge density of SiO_2_ nanoparticles with varying sizes (38), which shows that the surface charge density is approximately proportional to the curvature of the nanoparticle (ρ∝1/d), as illustrated by Fig. 2 *C*. When the elastic LH2 plate interacts with a charged spherical nanoparticle, the Coulombic interaction between them would induce two distinct types of deformation: in-plane elliptical deformation and out-of-plane bending deformation, as depicted in Fig. 3.

In solution, it has been demonstrated that free LH2, surrounded by a monolayer of detergent molecules, adopts an elliptical shape (26). When absorbed on the charged curved surface of the nanoparticles, the initial elliptical LH2 plate can be restored to a circular plate depending on the size of the particle. When the diameter of nanoparticle d is larger than a critical diameter $d_{c}$, the in-plane component of Coulombic interaction $F_{∥}\left(\theta_{1}\right)$ at the end of the major axis is larger than $F_{∥}\left(\theta_{2}\right)$ at the minor axis (i.e., $F_{∥}\left(\theta_{1}\right)>F_{∥}\left(\theta_{2}\right)$); the imbalance between the two forces would further drive the elliptical LH2 toward a circular shape until $F_{∥}\left(\theta_{1}\right)=F_{∥}\left(\theta_{2}\right)$. Through the derivation in supporting text, the critical size $d_{c}$ is given by

(1) $$d_{c}=a_{0}{\left[\left({\left(b_{0}/a_{0}\right)}^{\frac{2}{3}}-{\left(b_{0}/a_{0}\right)}^{2})/(1-{\left(b_{0}/a_{0}\right)}^{\frac{2}{3}}\right)\right]}^{1/2}\text{,}$$

where the major axis lengths $a_{0}$ = 11.0 nm and the minor axis lengths $b_{0}$ = 8.5 nm, based on small-angle X-ray scattering analysis of detergent-shelled LH2s for *Rba. sphaeroides* in solution (26). Accordingly, $d_{c}$ is calculated as 13.7 nm (Fig. S5). It can be inferred that LH2 would maintain its circular symmetry when the particle size is larger than $d_{c}$.

**Figure 4:**
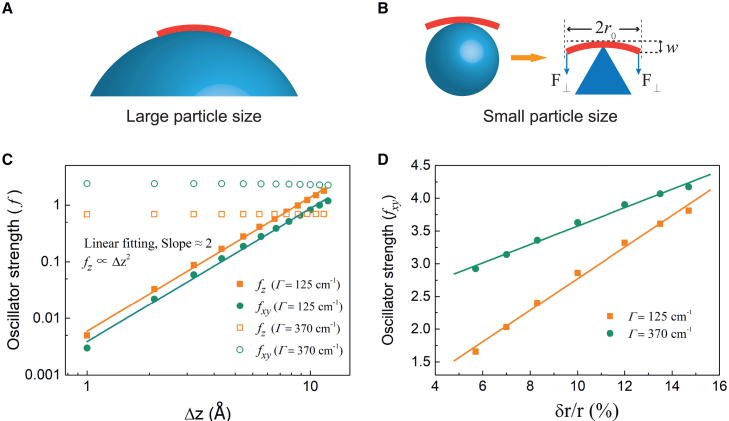
Schematic diagrams illustrating the way of different bending deflection on nanoparticles of either large or small size and calculated oscillator strength of the lowest excitonic state versus the deformation parameters. (*A*) LH2 (*thick red line*) fits the nanoparticle surface completely for large particle sizes. (*B*) LH2 contacts the particle surface incompletely for small particle sizes, where only the central part of the LH2 plate is supported by the nanoparticle represented by the triangle, and the edges of the plate experience an applied shear force. $r_{0}$, the radius of the LH2 plate; *w*, bending deflection. (*C*) Calculated oscillator strength components in *z* axis ($f_{z}$) and *xy* plane ($f_{xy}$) versus the vertical bending displacement Δz with two different random disorders of site energy Γ = 125 and 370 cm^−1^, respectively. Logarithmic coordinates are used. (*D*) Oscillator strength component in *xy* plane ($f_{xy}$) for elliptical deformation versus the radial deformation $\delta r/r_{0}$ at two different random disorders of site energy Γ = 125 and 370 cm^−1^, respectively.

The Coulombic interaction also causes out-of-plane deformation of LH2. When the particle size is large enough, it is plausible to assume that a slight deformation of LH2 would fit perfectly on the silica sphere, aligning its curvature with that of the nanoparticle surface, as shown in Fig. 4 *A*. In the extreme case of infinitely large nanoparticles (d→∞), the vertical deflection Δz approaches zero. The relation between Δz and d can be approximated as

(2) $$\Delta z\approx \frac{r_{0}^{2}}{2d}\;\propto \;\frac{1}{d}\text{,}$$

where $r_{0}$ is the radius of the LH2 ring.

As nanoparticle size decreases, the bending stiffness of LH2 hinders its yielding to match the curvature of the nanoparticles. Consequently, an elastic plate deflection model is proposed under these conditions, as illustrated in Fig. 4 *B*. In this model, the central part of the LH2 plate is supported by a fulcrum, while the edge of the plate experiences an applied shear force $q=F_{⊥}/L$. Here, $F_{⊥}$ is the vertical Coulombic force component and L is the circumference of the LH2 ring. It is assumed that the deflections of the plate (w) are small, i.e., $w\ll 2r_{0}$. Then, the deflection w of an isotropic elastic plate satisfies the Lagrange equation

(3) $$D\nabla^{4}w=0\text{,}$$

where D is the flexural rigidity of the plate (47). By solving the differential Eq. 3, we derived the vertical deformation for small particle size as

(4) $$\Delta z=w_{\max}\propto \rho /{\left(1+4r_{0}^{2}/d^{2}\right)}^{3/2}\text{,}$$

where ρ is the surface charge density of the silica nanoparticle. The detail derivation is given in the supporting material.

### The excited-state lifetime of B850 in distorted LH2 as an indicator of its structural deformation revealed by the excitonic theory

We will show that the lifetime of B850 (τ) can be used as a quantitative indicator of LH2 deformation, which is also directly related to its energy transfer efficiency. To establish a quantitative correlation between LH2 deformation and the B850 excited-state lifetime, the dipole oscillator strength (f) of the lowest excitonic state of the deformed B850 ring, which is directly proportional to the radiative decay rate, is calculated.

The system can be described by the Hamiltonian (48,49)

(5) $$H=\sum_{n=1}^{18}E_{n}|n\rangle \langle n|+\sum_{n=1}^{18}\sum_{m=1}^{2}t_{n,n+m}[|n\rangle \langle n+m|+|n+m\rangle \langle n|]\text{,}$$

where $E_{n}$ represents the site energy of *n*^*th*^ individual BChl *a* molecule and $t_{n,n+1}$ and $t_{n,n+2}$ are the nearest- and second-nearest-neighboring interaction*,* respectively. The local excited states are denoted by |n⟩ and |n+m⟩, where m = 1 or 2. The coherent one-exciton eigenfunction of the Hamiltonian (50) is expressed as $|k\rangle =\sum_{n=1}^{18}c_{kn}|n\rangle$, with corresponding coefficients $c_{kn}$. The dipole oscillator strength ($f_{k}$) of the excitonic state |k⟩ is given by $f_{k}={\vec{\mu }}_{k}^{*}\cdot {\vec{\mu }}_{k}$, where ${\vec{\mu }}_{k}$ is obtained from $\sum_{n=1}^{18}c_{kn}{\vec{\mu }}_{n}$ and ${\vec{\mu }}_{n}$ represents the transition dipole moment for the $Q_{y}$ band of *n*^*th*^ BChl *a* (22,49,51).

To describe the spherical deformation, the planar regions of LH2 were mapped onto spherical patches using a length-preserving transformation, and the atomic coordinates of the LH2 from *Rhodopseudomonas acidophila*, obtained from the PDB (PDB: 1NKZ), are used as basis for the calculation of ninefold symmetric LH2 (Fig. S6). An initial elliptical deformation with a magnitude of $\delta r/r_{0}=5.7\%$ (correlated disorder) was applied to match the single molecular spectroscopic features of free LH2 (49). Random disorder was also considered, and two typical values of random disorder, Γ = 125 and 370 cm^−1^ (24,52), were employed in the calculations, with the assumption that the random and correlated disorders are independent. All other parameters required to calculate f have been reported elsewhere (49).

Fig. 4, *C* and *D*, presents the calculated oscillator strengths of the lowest excitonic state as a function of the two deformation parameters, Δz for bending deflection and $\delta r/r_{0}$ for elliptical deformation, at the two different Γ values. The results reveal that the out-of-plane oscillator strength component ($f_{z}$) is proportional to the square of the bending deflection, fitted by

(6) $$f_{z}\;\propto \;{\Delta z}^{2}\text{.}$$

Equation 6 holds regardless of the selected Γ values. On the other hand, the in-plane oscillator strength component ($f_{xy}$) is nearly independent of the bending deformation, suggesting that the vertical bending and in-plane deformation can be treated independently. The calculated oscillator strength component in the *xy* plane ($f_{xy}$) shows a linear relationship with the elliptical deformation ($\delta r/r_{0}$). Obviously, when $d>d_{c}$, $\delta r/r_{0}=0$, and only the change in bending-induced oscillation strength is involved.

The experimentally measured decay lifetime τ includes contributions from both radiative decay rate ($k_{r}$) and nonradiative decay rates ($k_{nr}$). In principle, the fluorescence quantum yield (Φ) of LH2 absorbed on silica nanoparticles (LH2@SiO_2_) can be measured to determine $k_{r}$ using the relation $\Phi =k_{r}/\left(k_{r}+k_{nr}\right)$. However, determining accurate Φ is challenging due to significant scattering effect SiO_2_ particles of large sizes. Our recent study shows that there exists a proportional relationship between radiative and nonradiative electronic coupling elements (53). The linear relationship, given by $k_{nr}\left(T\right)\approx \left(k_{nr}^{0}/k_{r}^{0}\right)k_{r}\left(T\right)+\left[A\left(T\right)-B\left(T\right)\right]k_{nr}^{0}$, has been experimentally verified for highly structurally symmetric fluorescent molecules with weak electron-phonon coupling like LH2 at given temperatures (*T*), where $k_{nr}^{0}$ and $k_{r}^{0}$ are the rate constants at T=0K, whereas A(T) and B(T) are coefficients related to the vibrational modes at *T* (53). Here, we further show that this linear relationship also holds true for LH2@SiO_2_ with varied particle size, as shown in Fig. S7. Therefore, we have $f\;\propto \;k_{r}+k_{nr}=1/\tau$. By combining Eqs. 2, 4, and 6, the relations between the inverse excited-state lifetime and the nanoparticle diameter d ($>d_{c}$) are obtained as

(7) $$1/\tau \;\propto \;1/d^{2}$$

for large particle sizes and

(8) $$1/\tau \propto \rho^{2}/{\left(1+4r_{0}^{2}/d^{2}\right)}^{3}$$

for small particle sizes.

### Comparison of theoretically predicted excited-state lifetime of deformed LH2 on silica particles with results of experimental measurement

**Figure 5:**
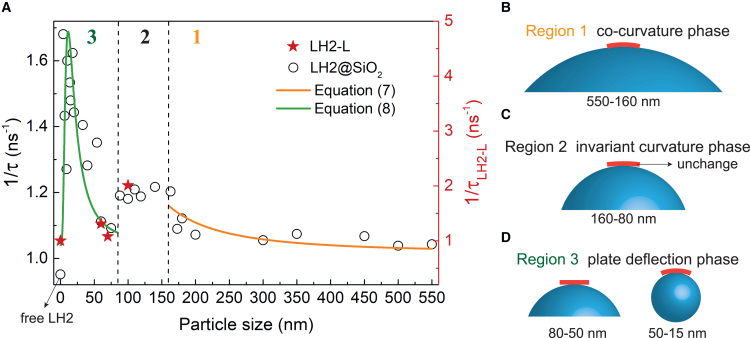
The measured B850 excited-state decay rate (1/τ) against the silica particle (or liposome) size and theoretical fitting. (*A*) The excited-state lifetime was measured by femtosecond time-resolved transient absorption of LH2@SiO_2_ (*circles*), together with LH2-Ls of varying sizes (*red pentagrams*). The curves for the size-dependent decay rate for LH2@SiO_2_ against the nanoparticle size can be divided into three regions: (*B*) the cocurvature phase in region 1 for larger particle sizes, (*C*) the invariant LH2 curvature phase in region 2 resulting from the bending stiffness of LH2 plate, and (*D*) the plate deflection phase in region 3 for small particle sizes, as represented by the model in Fig. 4*B*.

Fig. 5 demonstrates the measured B850 excited-state decay rate (1/τ) of LH2@SiO_2_ from *Rba. sphaeroides* against the size of the nanoparticles, and the corresponding decay kinetics of LH2@SiO_2_ are shown in Fig. S8. The size range of interest is from 15 to 550 nm, where LH2 assumes a circular symmetry in plane. Three distinct deformation stages are observed in Fig. 5 *A*. Region 1 (550–160 nm): in this larger size region, the bending deformation of LH2 aligns well with Eq. 7 of cocurvature deformation represented by the orange solid curve. This alignment indicates that for large particle sizes with small curvature, LH2 conforms effectively to the curved surface. Region 2 (160–80 nm): the lifetime remains relatively constant with changes in particle size, which has not been predicted in theory. This fact suggests that the deformation remains unchanged due to the bending stiffness of LH2, indicating LH2’s ability to resist the electrostatic forces from charged nanoparticles. This region implies an intrinsic curvature diameter of approximately 160 nm for LH2. Previous molecular dynamics simulations show that LH2 assemblies containing seven LH2s for sparse arrangement correspond to a curvature diameter of ∼160 nm, with a tilt angle of 5.4° between the two closely contacted LH2s (54), which is consistent with our findings. Region 3 (80–15 nm): as the particle size decreases to around 80 nm, a sudden decrease in 1/τ is observed, indicating a significant alleviation in LH2 deformation. This abrupt change suggests that the LH2 plate almost returns to its near-flat structure due to the increased separation distance between the nanoparticle surface and the LH2 plate’s edge. The electrostatic attraction becomes insufficient to dominate over the resilience of LH2, causing LH2 to revert to a near-flat form. The experimental 1/τ data in this region can be fitted with Eq. 8, as illustrated by the model in Fig. 4 *B*. Moreover, the increasing LH2 deformation with decreasing particle size in region 3 is attributed to the higher charge density of smaller silica nanoparticles, resulting in a higher vertical Coulombic force component ($F_{⊥}$) (Fig. S9).

Additionally, the measured decay rates of LH2-Ls ($1/\tau_{\text{LH}2-L}$) against the average size of the liposomes (60, 70, and 100 nm) are also plotted in Fig. 5 (*red pentagrams*). The measured lifetimes for all LH2@SiO_2_ and LH2-L samples are given in Table S1. Notably, the observed curvature-induced changes in LH2 lifetime in liposomes exhibited a consistent trend with those observed for LH2@SiO_2_ of similar sizes. This consistency reinforces the experimental approach of substituting liposomes with silica nanoparticles. In our liposome samples, the protein/lipid molar ratio was approximately 1:4000. We employed low excitation energy (8.5 × 10^9^ photons pulse^−1^ cm^−2^) and frequency (100 kHz) to prevent singlet-triplet and singlet-singlet annihilations within the LH2s (55).

## Discussion

Our results clearly reveal that LH2 restored its original circular and flat form within a specific size range of approximately 50–80 nm, which coincides with the optimized size distribution region found in ICM vesicles of photosynthetic bacteria. Within this size range, there is a distinct advantage in terms of the overall light conversion efficiency of a whole photosynthetic bacterial cell, particularly when adapting to low light intensities. The vesicles within this strategic evolutionary size range are small enough to maintain a larger effective light-collecting membrane area while preserving an undistorted LH2 structure, crucial for optimal EET efficiency.

In this research, we have presented evidence that quantum effects in the structural design principles of LH2 at the nanoscale (around 10 nm) regulate the size range of macro-sized ICM vesicles, with diameters falling into the region of 50–80 nm, where LH2 exhibits bending stiffness, highlighting its evolved structural robustness and superiority. As a result, the optimized vesicle size is regulated by quantum design principles for the construction of LH2s ideal for energy storage and transfer.

## Acknowledgments

This work was financially supported by the 10.13039/501100001809National Natural Science Foundation of China (grant no. T2350011) and the Major Research Plan of the National Natural Science Foundation of China (grant no. 92353000).

## Author contributions

Conceptualization, Y.W. and Y.Z.; formal analysis, Y.Z., Q.C., Y.Y., and Y.W.; methodology, Y.W. and Y.Z.; investigation, L.D., M.C., Y.Z., L.P., P.W., and H.C.; visualization, Y.Z.; funding acquisition, Y.W.; project administration, Y.W.; supervision, Y.W.; writing – original draft, Y.Z. and Y.W.; writing – review & editing, Y.W. and J.Z.

## Declaration of interests

The authors declare no competing interests.

## Supporting citations

References (56,57,58,59,60) appear in the supporting material.

## Supporting material

Document S1. Figures S1–S9, Table S1, and supporting text

Document S2. Article plus supporting material
