# Supplementary material for: Macroscale optimal size of ICM vesicles regulated by quantum design principle in LH2 structure
Source: Biophys J. 2025 Jun 7;124(14):2317–26. doi: 10.1016/j.bpj.2025.06.004 (PMC12414671; doi:10.1016/j.bpj.2025.06.004)
Supplement: Document S1. Figures S1–S9, Table S1, and supporting text [file mmc1.pdf]

Biophysical Journal, Volume *124*

Supplemental information

Macroscale optimal size of ICM vesicles regulated by quantum design principle in LH2 structure

Ying Zhang, Qianjin Chu, Luchao Du, Yugui Yao, Hailong Chen, Peng Wang, Jianping Zhang, Mingqing Chen, Lingfeng Peng, and Yuxiang Weng

Supplemental Information for

Macroscale Optimal Size of ICM Vesicles Regulated by Quantum Design Principle in LH2 Structure

Authors: Ying Zhang[1,2], Qianjin Chu[3], Luchao Du[3], Yugui Yao[4], Hailong Chen[1,2,5], Peng Wang[6], Jianping Zhang[6], Mingqing Chen[6], Lingfeng Peng[5], Yuxiang Weng[1,2,5]*

[1]Laboratory of Soft Matter Physics, Institute of Physics, Chinese Academy of Sciences; Beijing, 100190, China.

[2]University of Chinese Academy of Sciences; Beijing, 100049, China.

[3]Institute of Physics, Chinese Academy of Sciences; Beijing, 100190, China.

[4]Beijing Institute of Technology; Beijing, 100081, China.

[5]Songshan Lake Materials Laboratory; Dongguan, 523808, China.

[6]Renmin University of China; Beijing, 100872, China.

*Corresponding author. Email: yxweng@iphy.ac.cn.

The PDF file includes:

Supplementary Text
Figs. S1 to S9
Tables S1

Supplementary Text

The similar shear force applied on LH2 for LH2 on silica particles and LH2 in vesicles for large size

When the size of ICM vesicles or silica particles is much larger than the radius of LH2 ($R \gg r_0$, R and r_0 are the radius of sphere and LH2 plate, respectively), we first analyze the shear force acting on LH2 embedded in vesicles. The Young-Laplace equation describes the pressure difference (ΔP) between two static fluid interfaces due to surface tension. Since the radius of curvature is positive for convex surfaces and negative for concave surfaces, the additional pressure always points toward the center of the sphere. According to the Young-Laplace equation, the pressure difference across the vesicle membrane is related to its curvature ($1/R$) by $\Delta P = \frac{2\gamma}{R}$, where γ is the surface tension (56). The resulting shear force applied on LH2 embedded in the vesicle can be expressed as

$$q = \frac{F_\perp}{L} \propto \frac{\Delta P \times \pi r_0^2}{2\pi r_0} = \frac{\gamma r_0}{R} \qquad \text{(S1)}$$

where $F_\perp$ is the stress force normal to the LH2 plate, L is the circumference of LH2 and r_0 is the radius of LH2. This shows that LH2 in the membrane experiences a stress inversely proportional to the vesicle radius.

Next, we examine the system where LH2 is embedded in a bilayer attached to rigid beads. The work of Bayerl *et al.* investigated the nuclear magnetic resonance (NMR) transverse relaxation times (τ_2) of spherical supported vesicles (SSVs) constituting of single phospholipid bilayers adsorbed onto glass beads (57). Their findings demonstrate a strong dependence of the transverse relaxation times on the curvature of the SSVs for diameters between 0.5 and 1.5 μm. The transverse relaxation time is related to the sphere radius R by the equation $\tau_2 = \frac{R}{6\mathcal{D}}$, where $\mathcal{D}$ is the diffusion constant (58). It has been shown that the relaxation time τ_2 is roughly inversely proportional to the bilayer viscosity (η), i.e. $\tau_2 \propto \frac{1}{\eta}$ (59). If we consider the lipid bilayer as an Newtonian liquid, the shear force can be written as $q = \eta \times \gamma'$ where γ' is the shear rate constant (60). Combining these relationships yields:

$$q \propto \frac{6\mathcal{D} \times \gamma'}{R}. \qquad \text{(S2)}$$

Finally, we consider LH2 adsorbed on a silica sphere (our system in this research) when $R >> r_0$. The surface charge density (ρ) on the silica sphere can be expressed as $\rho \propto \frac{1}{d}, d = 2R$. (38) The total charge of the silica sphere is $4\pi R^2 \times \rho \propto \pi R$. Owing to the symmetry and uniform surface charge distribution, the silica nanoparticle can be approximated as an equivalent point charge ($4\pi R^2 \rho$) at the sphere's center. The Coulombic attraction force between LH2 and the silica sphere is $F \propto \frac{\sigma \times \pi R}{4\pi\varepsilon_0 R^2} = \frac{\sigma}{4\varepsilon_0 R}$, where ε_0 is the dielectric constant and σ is the total charge on the LH2 ring. Then the shear force on LH2 at this situation is

$$q = \frac{F_\perp}{L} \propto \frac{\sigma \varepsilon_0{}^{-1} r_0{}^{-1}}{R}. \qquad \text{(S3)}$$

Obviously, the shear forces on LH2 in all three systems (vesicles, supported bilayers by beads, and silica particles), are inversely proportional to the sphere radius. This indicates that for sufficiently large R, the shear forces applied on LH2 in vesicles and on silica particles are similar.

Derivation of the critical nanoparticle size for an elliptical LH2 restoring to a circular form

The Coulombic attractive interactions, as depicted by Fig. S5A, between the end of major axis or minor axis of LH2 ellipse and the charged silica nanoparticle can be written as $F(\theta_1) =$

$\frac{\pi d^2\rho\sigma/L}{4\pi\varepsilon_0 r_1^2} = \frac{d^2\rho\sigma}{\varepsilon_0 L(d^2+a_0^2)}$ or $F(\theta_2) = \frac{\pi d^2\rho\sigma/L}{4\pi\varepsilon_0 r_2^2} = \frac{d^2\rho\sigma}{\varepsilon_0 L(d^2+b_0^2)}$, respectively. θ_1 and θ_2 are defined as the angles between xy-plane and the line connecting the sphere center and the endpoint of the major axis or minor axis of the LH2 ellipse. r_1 and r_2 are the distances between the sphere center and the endpoint of the major axis or minor axis of the LH2 ellipse. Here d is the silica nanoparticle diameter; ρ is the surface charge density of nanoparticles; σ is the total charge on LH2 ring at the plate's edge as stated above; L is the perimeter of the LH2 ring and ε_0 is the vacuum dielectric constant. The major axis lengths a_0=11.0 nm and the minor axis lengths b_0=8.5 nm of elliptical LH2 plate in solution were determined by small angle X-ray scattering (SAXS) analysis of detergent-shelled LH2 complexes for *Rhodobacter* (*Rba.*) *sphaeroides* (26). The surface charge density is approximately proportional to the curvature of the silica nanoparticle ($\rho \propto 1/d$) (38). Then the according in-plane Coulombic attractive force components are given by

$$F_{\parallel}(\theta_1) = F(\theta_1)\cos(\theta_1) \propto \frac{da_0}{(d^2+a_0^2)^{3/2}} \qquad \text{(S4-1)}$$

and

$$F_{\parallel}(\theta_2) = F(\theta_2)\cos(\theta_2) \propto \frac{db_0}{(d^2+b_0^2)^{3/2}}. \qquad \text{(S4-2)}$$

These two in-plane Coulombic interaction components with varying particle sizes calculated through Eq. S4 are presented in Fig. S5B. There exists a critical diameter d_c such that $F_{\parallel}(\theta_2) > F_{\parallel}(\theta_1)$ when the particle size is smaller than d_c, but $F_{\parallel}(\theta_1) > F_{\parallel}(\theta_2)$ when the particle size is larger than d_c. By solving the equation $F_{\parallel}(\theta_1)|_{d=d_c} = F_{\parallel}(\theta_2)|_{d=d_c}$, the critical size d_c is calculated as

$$d_c = a_0\sqrt{\frac{(b_0/a_0)^{\frac{2}{3}}-(b_0/a_0)^2}{1-(b_0/a_0)^{\frac{2}{3}}}}. \qquad \text{(S5)}$$

For the elliptical LH2 plate absorbed on charged curved surface, when $F_{\parallel}(\theta_1) > F_{\parallel}(\theta_2)$, the non-equilibrium between the two forces would drive an elliptical LH2 plate towards a circular shape until $F_{\parallel}(\theta_1) = F_{\parallel}(\theta_2)$.

The derivation of the relationship between the bending deflection Δz of the LH2 plate and the size of nanoparticle

Considering the deformation of a plate under an applied shear force $q = F_{\perp}/L$ at a point of the plate's edge, where the total applied force $F_{\perp}$ is the out-of plane Coulombic interaction components and L is the circumference. The central part of the LH2 plate is supported by the nanoparticle. Only small deflection is considered, i.e., $w \ll 2r_0$, where w represents the deflection of the plate and r_0 is the radius of undeformed plate. The deflection w of an isotropic elastic plate satisfies the Lagrange equation (47)

$$D\nabla^4 w = 0 \qquad \text{(S6)}$$

where $D = \frac{Eh^3}{12(1-\nu^2)}$ is the flexural rigidity of the plate, E is Young's modulus, ν is the Poisson ratio, and h is the thickness of the plate. The general solution for deflection in Eq. S6 is

$$w = C_1 + C_2 r^2 + C_3 \ln\frac{r}{r_0} + C_4 r^2 \ln\frac{r}{r_0} \qquad \text{(S7)}$$

where r is the polar coordinate, and C_k (k=1,2,3,4) are coefficients which can be determined by the boundary conditions to Eq. S6: (i) $w(r=0)=0$ at the center of the plate; (ii) the bending moment $M_r(r=r_0)=0$ at the plate edge; (iii) the applied shear force q at the plate edge. With boundary condition (i), we have C_1=0, C_3=0, thus w reduces to

$$w = C_2 r^2 + C_4 r^2 \, ln\frac{r}{r_0}. \qquad \text{(S8)}$$

Using boundary condition (ii), $M_r(r = r_0) = -D\left(w'' + \frac{v}{r}w'\right)|_{r=r_0} = 0$, we have

$$2C_2 + 3C_4 + v(2C_2 + C_4) = 0. \qquad \text{(S9)}$$

The boundary condition (iii) which arises from the balance of forces, i.e. $q = -D\frac{\partial}{\partial r}(\nabla^2 w)|_{r=r_0}$, will lead to

$$q = -2DC_4/r_0. \qquad \text{(S10)}$$

Combining Eq. S8, Eq. S9 and Eq. S10, finally, we have $C_1 = 0$, $C_2 = \frac{(3+v)qr_0}{4(1+v)D}$, $C_3 = 0$, $C_4 = -\frac{qr_0}{2D}$. Substituting the coefficients into Eq. S7, the solution of the deflection is $w = \frac{(3+v)qr_0}{4(1+v)D}r^2 - \frac{qr_0}{2D}r^2 \, ln\frac{r}{r_0}$. Therefore the maximum deflection can be realized at the edge, i.e.

$$w_{max} = w(r = r_0) = \frac{(3+v)F_\perp {r_0}^3}{4(1+v)DL}. \qquad \text{(S11)}$$

When $d > d_c$, the whole Coulombic attractive interaction between the plate and the charged nanoparticle can be written as $F_{Coul} = \frac{\pi d^2 \rho\sigma}{4\pi\varepsilon_0[(d/2)^2+(r_0)^2]} = \frac{d^2\rho\sigma}{\varepsilon_0(d^2+4r_0^2)}$, σ is the total charge of the LH2 ring. Then we have

$$F_\perp = F_{Coul}\sin\theta = \frac{\rho\sigma}{\varepsilon_0(1+4r_0^2/d^2)^{3/2}} . \qquad \text{(S12)}$$

Substituting Eq. S12 into Eq. S11, we find the maximum deflection of the plate to be

$$w_{max} = \frac{(3+v){r_0}^2}{8\pi(1+v)D}\frac{\rho\sigma}{\varepsilon_0(1+4r_0^2/d^2)^{3/2}}. \qquad \text{(S13)}$$

Finally, when v and D are fixed, we obtain the Eq. 4 in the main text

$$\Delta z = w_{max} \propto \frac{\rho}{(1+4r_0^2/d^2)^{3/2}}. \qquad \text{(S14)}$$

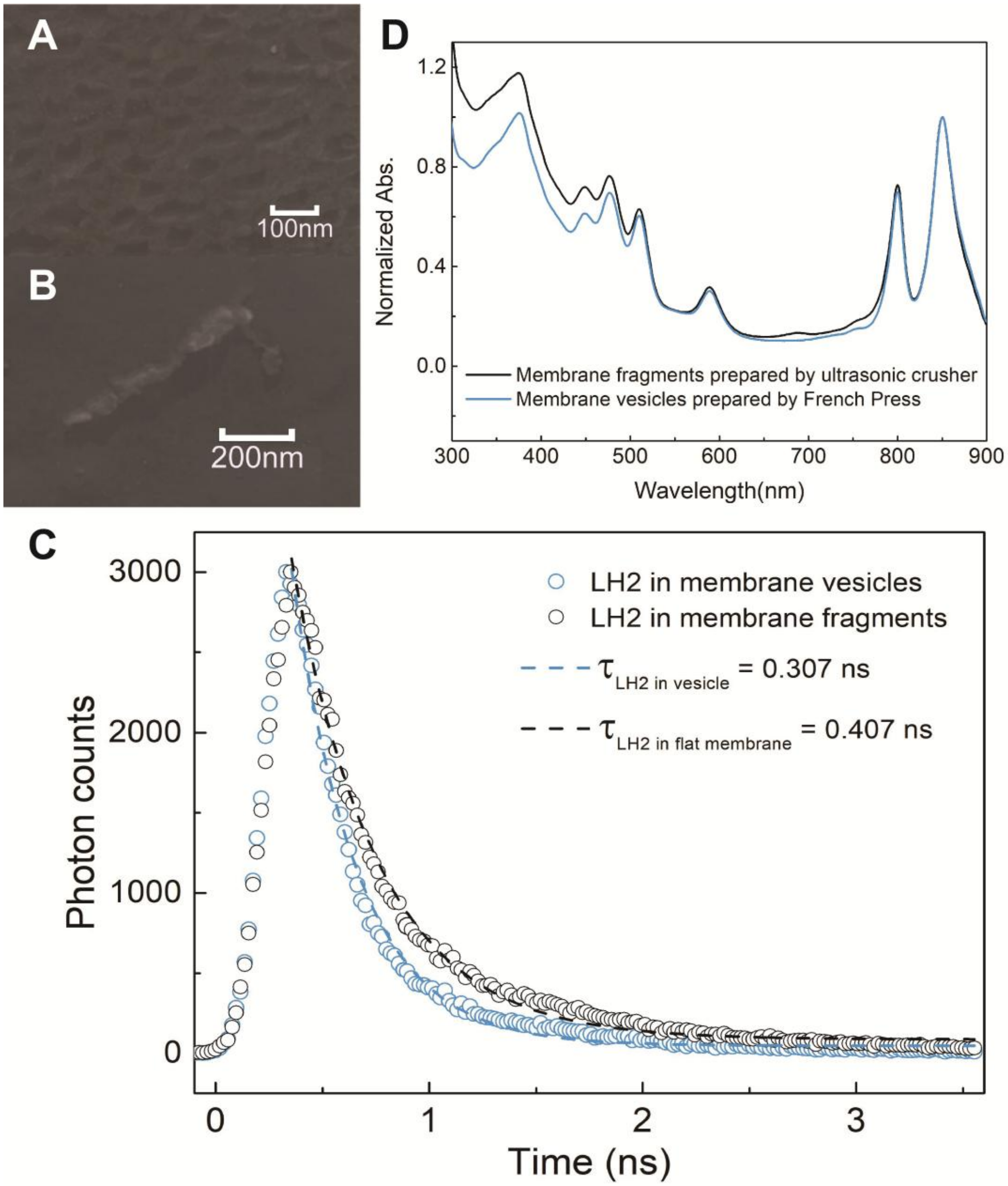

Fig. S1. The fluorescence decay kinetics of LH2 from *Rba. sphaeroides* observed in curved and flat membranes. Typical electron micrographs of Pt/C shadowed membrane of (A) vesicles prepared by disruption cells with French Press and (B) membrane fragments prepared by ultrasonic crusher. (C) The fluorescence decay kinetics were measured by the time-correlated single-photon counting (Edinburgh Instruments) obtained for the LH2 excited state in photosynthetic vesicles and membrane fragments. (D) The UV-visible absorption spectrum of the curved and flat photosynthetic membrane.

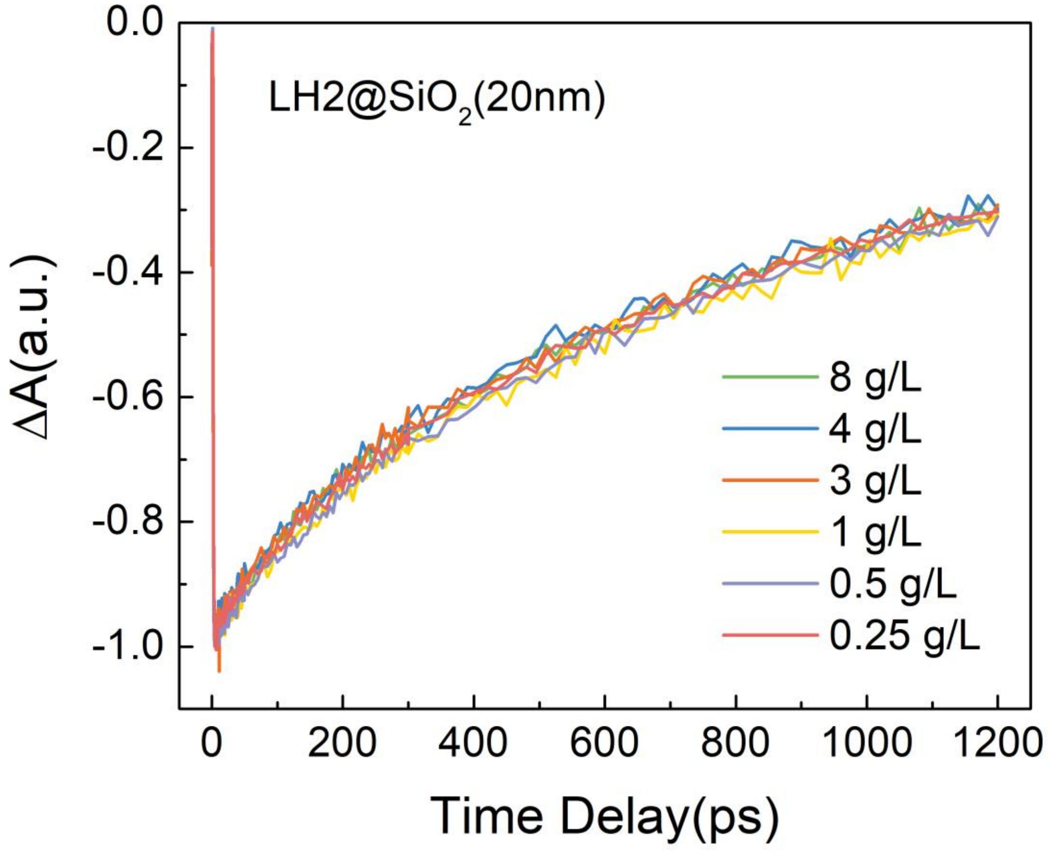

Fig. S2. Transient bleaching recovery kinetics of B850 in LH2 adsorbed on silica nanoparticles. LH2 from *Rb. sphaeroides* 2.4.1 were adsorbed onto 20 nm silica nanoparticles at various colloidal concentrations of 0.25, 0.5, 1.0, 3.0, 4.0 and 8.0 g/L, as indicated by the solid lines of different colors shown in the labels.

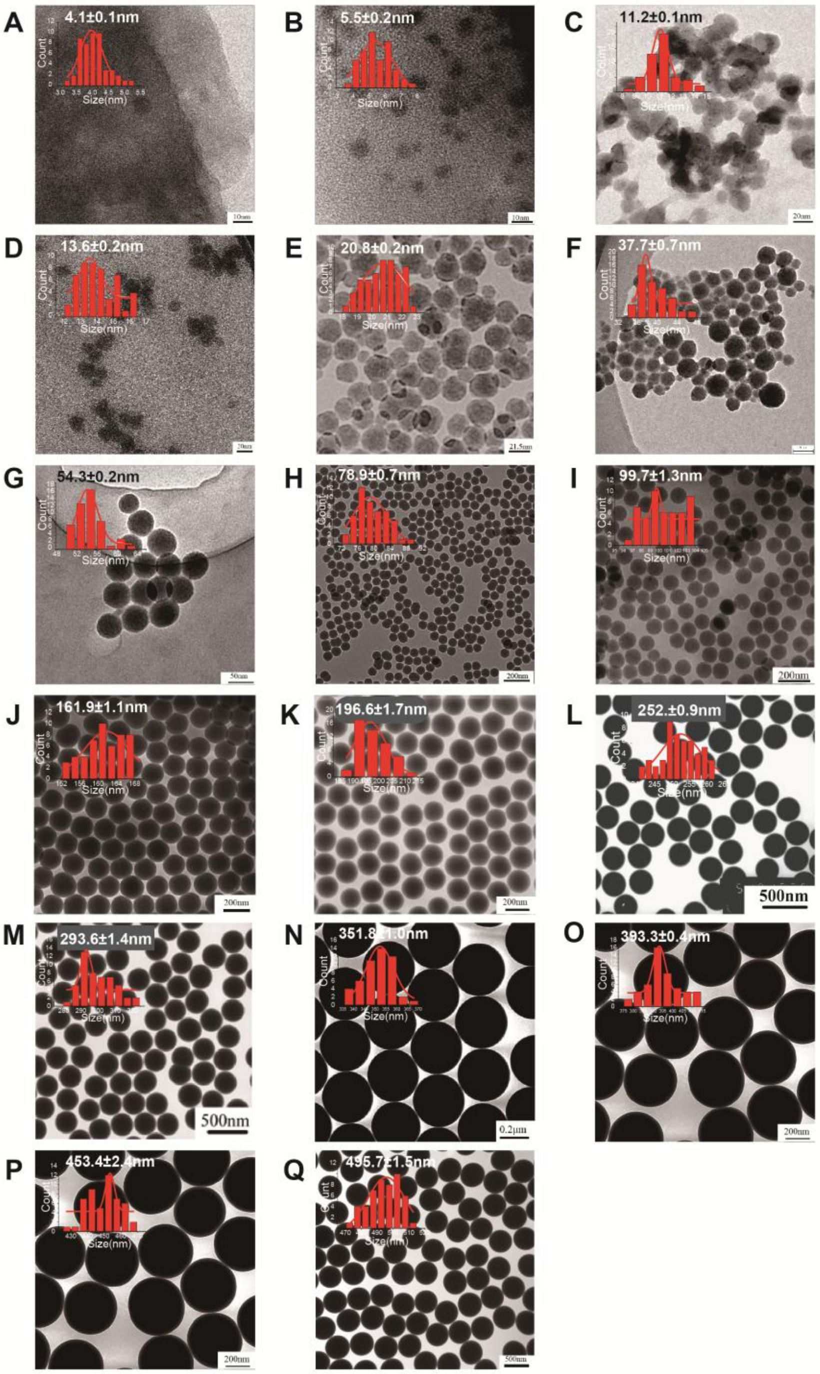

Fig. S3. TEM images for all the silica particles of varied size. (A) 4.1 nm; (B) 5.5 nm; (C) 11.2 nm; (D) 13.6 nm; (E) 20.8 nm; (F) 37.7 nm; (G) 54.3 nm; (H) 78.9 nm; (I) 99.7 nm; (J) 161.9 nm; (K)196.6 nm; (L) 252.1 nm; (M) 293.6 nm; (N) 351.8 nm; (O) 393.3 nm; (P) 453.4 nm; (Q) 495.7 nm. The graphic insets are the statistic column diagrams for the corresponding nanoparticles size distribution.

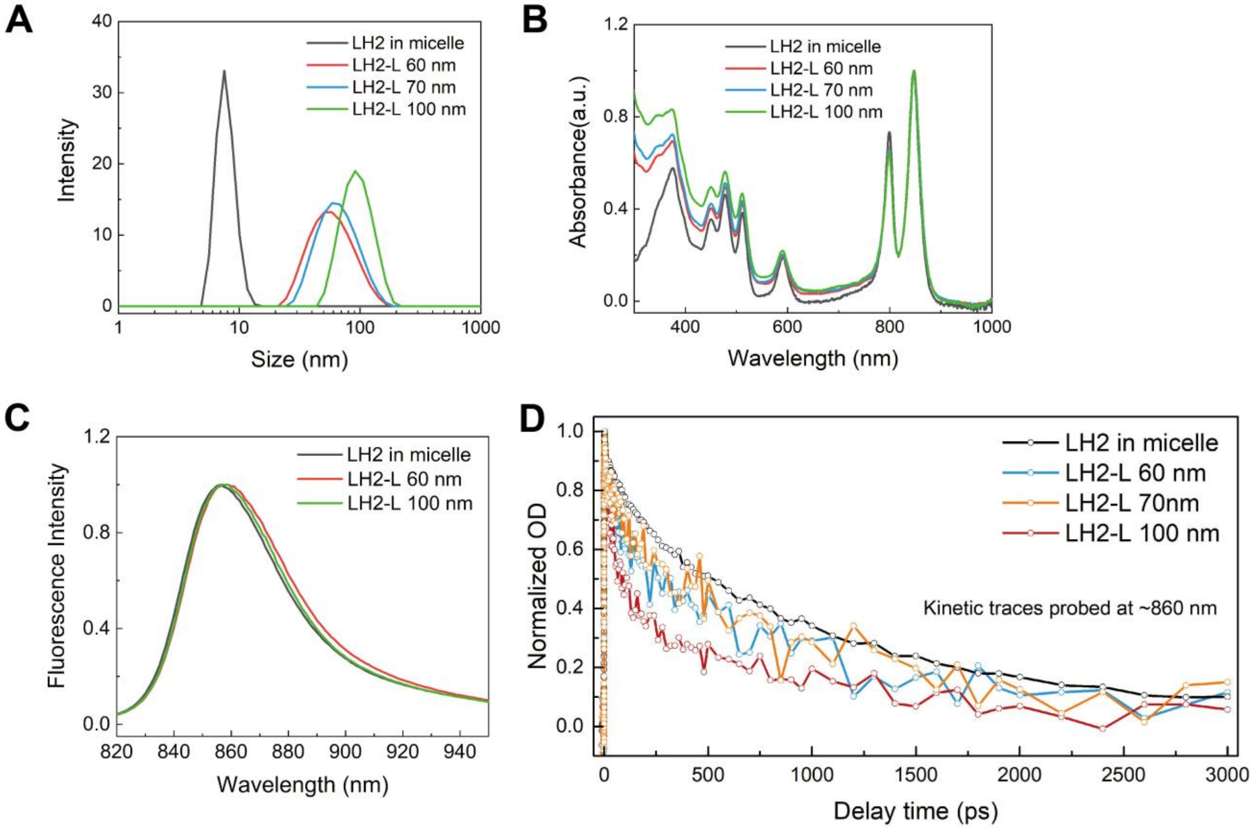

Fig. S4. The decay kinetics of LH2 from *Rba. sphaeroides* embedded in lipid vesicles with varying size measured by transient absorption. (A) Vesicle sizes of LH2-L measured by dynamic light scattering (DLS). The average sizes for LH2-L are 60±40 nm, 70±45 nm, 100±60 nm. (B) The UV absorption spectra of LH2-L samples. The spectra were normalized at B850 absorption maxima. (C) The fluorescence spectra of B850 in LH2-L samples, normalized at absorption maxima. The fluorescence peak for LH2 in liposomes is red-shifted and broadened compared to free LH2. (D) The bleaching kinetics of LH2-L at ~860 nm by transient absorption.

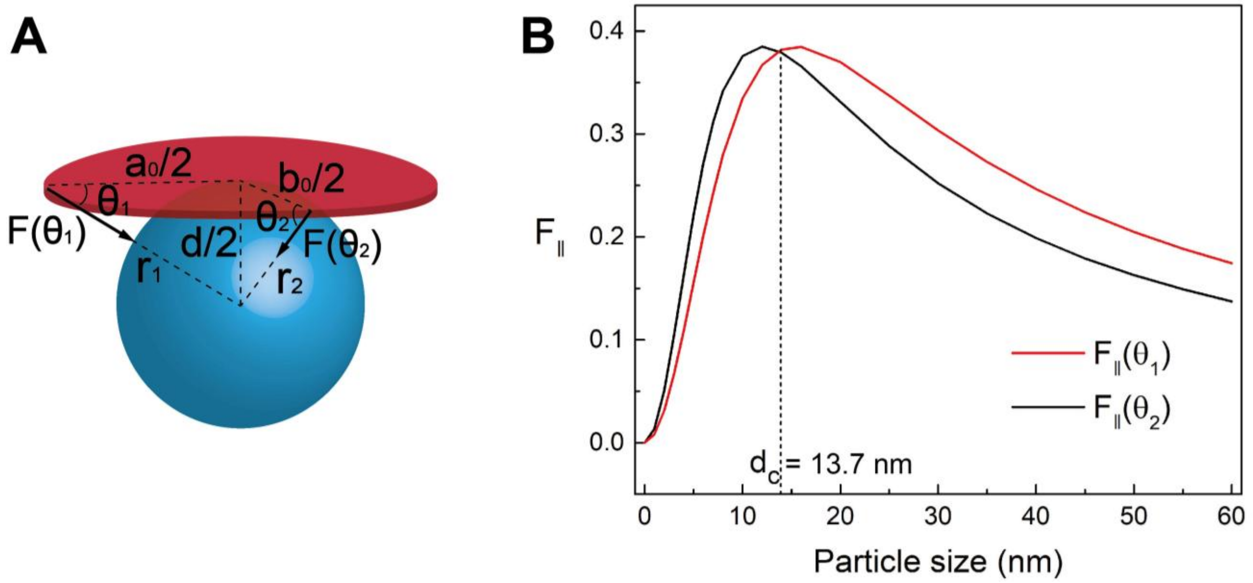

Fig. S5. The in-plane components of the Coulombic forces $F_{\parallel}$ against the particle size. (A) Schematic representation of the force on LH2 when LH2 interacts with the charged silica nanoparticles. (B) The calculated in-plane Coulombic interaction components $F_{\parallel}(\theta_1)$ and $F_{\parallel}(\theta_2)$ at the end of the major axis and the minor axis with varying particle sizes, respectively.

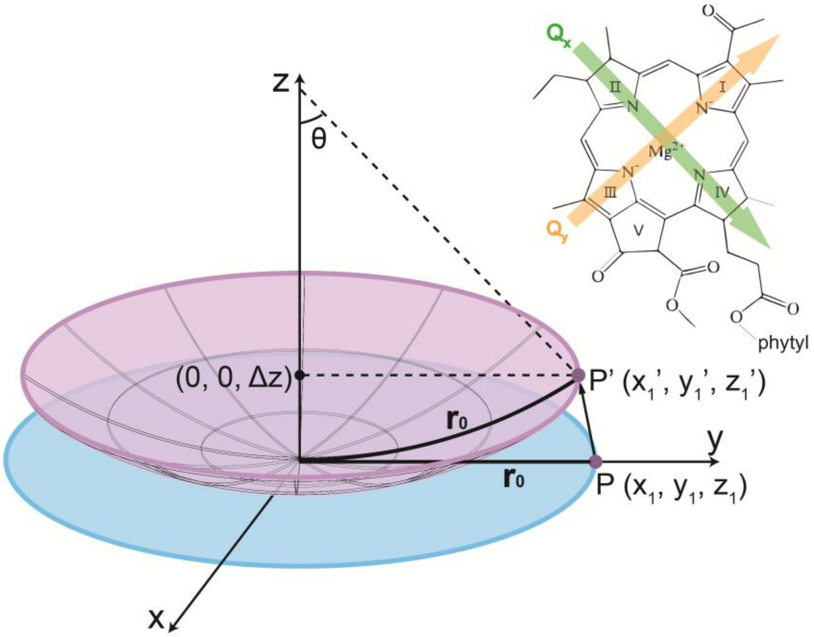

Fig. S6. Length-preserving transformation of mapping planar regions to spherical patches. The inset graph shows the structure of BChl *a* and its transition dipoles (23). For each magnesium or nitrogen atom on the B850 ring with a radius of r_0, there is one local plane which contains the atom and is parallel to the xy-plane. We use the point at which the z-axis passes through the local plane as the fixed point to deform the planar region to the spherical patch. The lengths of the line on the plane and the arc on the spherical surface between the atom and the fixed point are assumed to be equal during the transformation, i.e., the radius on the plane keeps the same length r_0 when mapped into the sphere as an arch. In one transformation, the spherical radii corresponding to all atoms are the same, and we use the maximal displacement (Δz) of the magnesium atoms on the ring as the marker of the transformation. After the deformation, the orientations of the Q_y transition dipoles of the BChl *a* are determined by the new coordinates of the N_I and N_{III} nitrogen atoms, and the BChl *a* molecules can be considered to be rigid during the deformation. For a point P (x_1, y_1, z_1) =(x_1, y_1, 0) in the original B850 ring in the plane, after the length-preserving transformation to the point P' on the sphere, the coordinates become P'(x_1', y_1', Δz), where $x_1' = \frac{\sqrt{r_0^2-\Delta z^2}}{r_0}x_1$, $y_1' = \frac{\sqrt{r_0^2-\Delta z^2}}{r_0}y_1$. The atomic coordinates of the LH2 are obtained from the structure of LH2 complex from *Rhodopseudomonas* (*Rps.*) *acidophila* through the Protein Data Bank (PDB ID: 1nkz). High-resolution structural information of LH2 from *Rba. sphaeroides* is reported and uploaded to the Protein Data Bank in 2021 by P. Qian *et al.* (21) Until then 1nkz is usually used as basis for the calculation of nine-fold symmetric LH2. The structure of the B850 ring pigment arrangement of LH2 is very similar in both strains. The Mg−Mg distance between paired BChls intradimer or interdimer is 9.3 or 9.2 Å, respectively, for LH2 in *Rba. sphaeroides*, while the intradimer and interdimer Mg−Mg distances are 9.5 and 9.0 Å, respectively, for LH2 in *Rps. acidophila* (21). These small differences have small effect on the calculation of the overall sum of Q_y transition dipoles across the B850 ring.

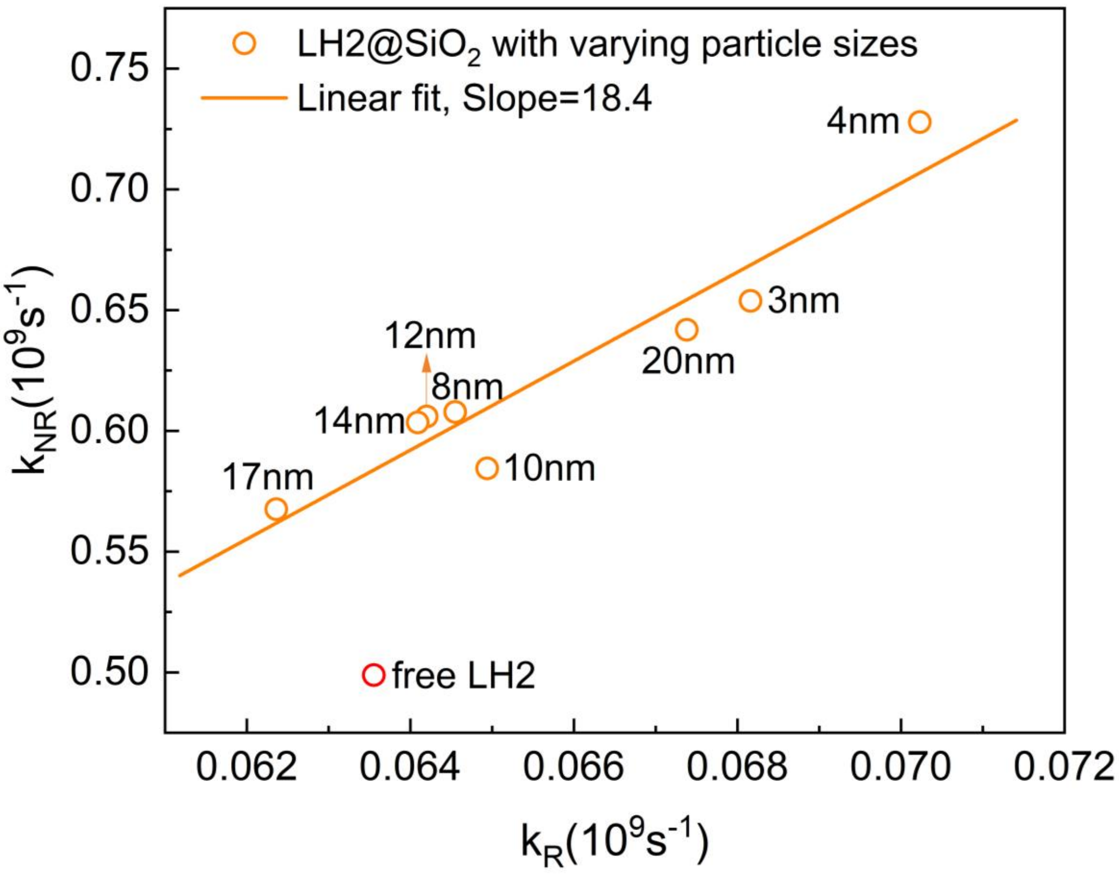

Fig. S7. The linear relationship between radiative and nonradiative decay rates in LH2@SiO$_2$ with varying silica nanoparticle sizes. The radiative and nonradiative decay rates are calculated from the experimentally measured lifetime (τ) and fluorescence quantum yield (Φ). The sizes of silica nanoparticles range from 3-20 nm, since larger particles lead to strong scattering effect, preventing precise measurement of Φ.

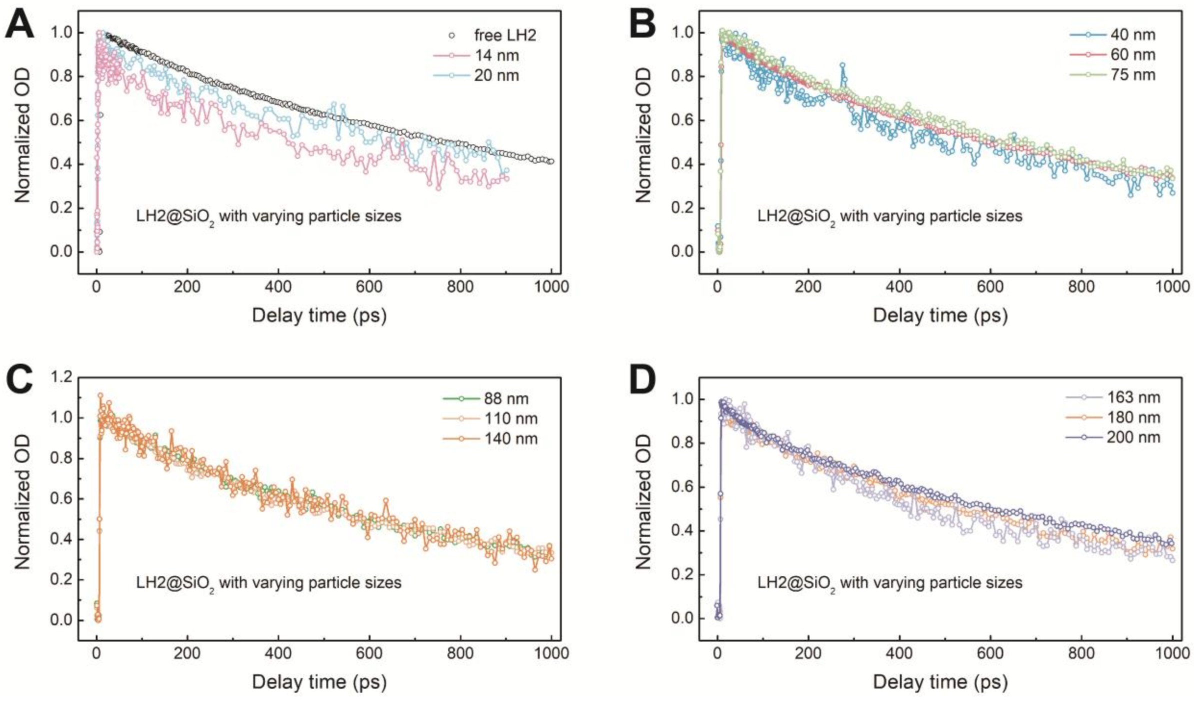

Fig. S8. The decay kinetics of LH2 from *Rba. sphaeroides* adsorbed on nanoparticles with varying sizes measured by transient absorption. The decay kinetics of LH2@SiO_2 for some typical groups of particle sizes are shown: (A) 0, 14, 20 nm in small part of region 3, where the smaller silica particles with higher surface charge densities induce larger deformation of LH2; (B) 40, 60, 75 nm in region 3, where it is shown that LH2 deformation is quite slight at 60 and 75 nm, indicating that the size range of 50-80 nm is the optimal region; (C) 88, 110, 140 nm in region 2, where LH2 deformation does not change with particle size due to the stiffness of LH2; (D) 163, 180, 200 nm in region 1, where the deflection of LH2 decreases with increasing particle size.

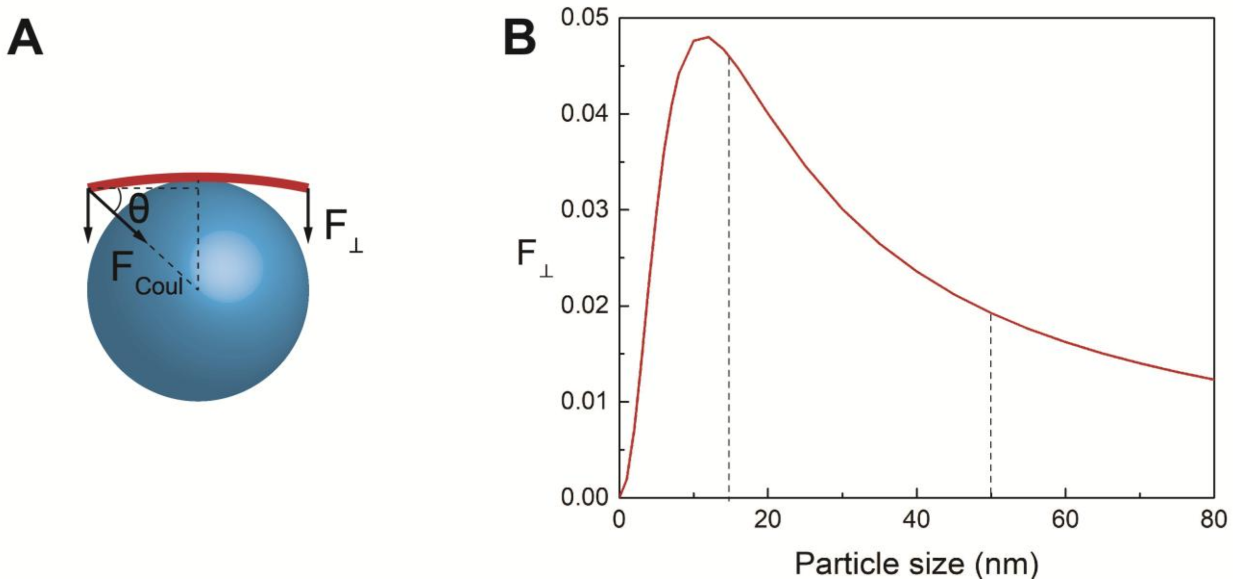

Fig. S9. The out-of-plane components of the Coulombic forces $F_{\perp}$ against the particle size. (A) Schematic representation of the shear force on LH2 plate for $d > d_c$. (B) The calculated out-of-plane Coulombic interaction components $F_{\perp}$ with varying particle sizes. The vertical dashed curves are at 15 and 50 nm.

LH2@SiO_2	Lifetime (ns)
0 nm (free LH2)	1.05 $\pm$ 0.006
4 nm	0.60 $\pm$ 0.009
6 nm	0.70 $\pm$ 0.009
9 nm	0.79 $\pm$ 0.01
10 nm	0.63 $\pm$ 0.011
14 nm	0.65 $\pm$ 0.014
15 nm	0.68 $\pm$ 0.009
18 nm	0.62 $\pm$ 0.0188
20 nm	0.69 $\pm$ 0.011
33 nm	0.71 $\pm$ 0.01
40 nm	0.78 $\pm$ 0.013
54 nm	0.74 $\pm$ 0.01
60 nm	0.90 $\pm$ 0.005
75 nm	0.92 $\pm$ 0.007
88 nm	0.84 $\pm$ 0.007
100 nm	0.85 $\pm$ 0.008
110 nm	0.83 $\pm$ 0.007
119 nm	0.84 $\pm$ 0.009
140 nm	0.82 $\pm$ 0.013
163 nm	0.83 $\pm$ 0.015
173 nm	0.92 $\pm$ 0.008
180 nm	0.90 $\pm$ 0.009
200 nm	0.93 $\pm$ 0.007
300 nm	0.95 $\pm$ 0.007
350 nm	0.93 $\pm$ 0.008
450 nm	0.94 $\pm$ 0.008
500 nm	0.96 $\pm$ 0.01
550 nm	0.96 $\pm$ 0.009

LH2-L	Lifetime (ns)
0 nm (free LH2)	1.01 $\pm$ 0.062
60 nm	0.77 $\pm$ 0.056
70 nm	0.93 $\pm$ 0.101
100 nm	0.50 $\pm$ 0.063

Table S1. The lifetimes of all LH2@SiO_2 and LH2-L samples measured by transient absorption. The kinetics for each sample is the average of at least three sets of measurements. The standard deviation is from the exponential decay fit.
